# Hemagglutinin Stability and Its Impact on Influenza A Virus Infectivity, Pathogenicity, and Transmissibility in Avians, Mice, Swine, Seals, Ferrets, and Humans

**DOI:** 10.3390/v13050746

**Published:** 2021-04-24

**Authors:** Charles J. Russell

**Affiliations:** Department of Infectious Diseases, St. Jude Children’s Research Hospital, 262 Danny Thomas Place, Memphis, TN 38105-3678, USA; charles.russell@stjude.org; Tel.: +1-901-595-5648

**Keywords:** influenza virus, hemagglutinin, virus fusion glycoprotein, animal model, ferret, transmissibility, adaptation, evolution, protein stability

## Abstract

Genetically diverse influenza A viruses (IAVs) circulate in wild aquatic birds. From this reservoir, IAVs sporadically cause outbreaks, epidemics, and pandemics in wild and domestic avians, wild land and sea mammals, horses, canines, felines, swine, humans, and other species. One molecular trait shown to modulate IAV host range is the stability of the hemagglutinin (HA) surface glycoprotein. The HA protein is the major antigen and during virus entry, this trimeric envelope glycoprotein binds sialic acid-containing receptors before being triggered by endosomal low pH to undergo irreversible structural changes that cause membrane fusion. The HA proteins from different IAV isolates can vary in the pH at which HA protein structural changes are triggered, the protein causes membrane fusion, or outside the cell the virion becomes inactivated. HA activation pH values generally range from pH 4.8 to 6.2. Human-adapted HA proteins tend to have relatively stable HA proteins activated at pH 5.5 or below. Here, studies are reviewed that report HA stability values and investigate the biological impact of variations in HA stability on replication, pathogenicity, and transmissibility in experimental animal models. Overall, a stabilized HA protein appears to be necessary for human pandemic potential and should be considered when assessing human pandemic risk.

## 1. Influenza Virology

Influenza viruses are enveloped, multisegmented, negative-sense RNA viruses of the family *Orthomyxoviridae*. Four of the seven genera (and species) of orthomyxoviruses are *Alphainfluenzavirus* (influenza A virus), *Betainfluenzavirus* (influenza B virus), *Gammainfluenzavirus* (influenza C virus), and *Deltainfluenzavirus* (influenza D virus). Influenza A viruses (IAVs) of great genetic diversity circulate in a reservoir of wild, aquatic birds [1,2]. Along with IAVs, influenza B viruses cause seasonal epidemics in humans and have been known to infect seals [3]. Influenza C viruses infect swine and humans, and the closely related influenza D viruses infect cattle and swine [4,5]. IAVs are more numerous, genetically diverse, circulate in more species, and have caused pandemics every twenty years or so over the last century. The focus of this review is IAVs and how the stability of the major surface antigen, the hemagglutinin (HA) protein, contributes to IAV host range, pathogenicity, and transmissibility. Starting with Section 6 (below), the biological impact of HA stability is reviewed in detail by subtype.

The eight RNA gene segments of IAV in order of size are PB2, PB1, PA, HA, NP, NA, M, and NS [6]. They are often numbered 1 (PB2) to 8 (NS) with the HA and neuraminidase (NA) genes that encode surface antigens considered segments 4 and 6, respectively. The polymerase complex (consisting of PB1, PB2, and PA) binds to the promoter region at the ends of each gene segment. The nucleoprotein (NP) encapsidates the RNA, and the M1 matrix protein interacts with the ribonucleoprotein (RNP), plasma membrane, and envelope glycoproteins to promote virus budding. The M2 ion channel protein allows proton flow to neutralize the secretory pathway during virus egress and to acidify entering, fused virions to allow uncoating of the viral gene segments and polymerase complex. Nonstructural accessory proteins include NS1 and other proteins such as PB1-N40, PA-X, PB1-F2, M42, NS3, PA-N155, and PA-N182 [1]. Wild, aquatic birds harbor IAVs of 16 HA and 9 NA antigenic subtypes [7]. H17N10 and H18N11 subtypes have been identified in bats [8,9]. Additionally, an H9N2 virus has been recovered from bats in Egypt [10]. A variety of subtypes have caused outbreaks and epidemics in domestic poultry and swine, and IAVs of the subtypes H1N1, H1N2, and H3N2 are currently endemic in swine [11,12,13].

## 2. Molecular Properties Contributing to In Vivo Phenotypes of IAVs

While multiple molecular properties contribute to the in vivo phenotypes of IAVs [14], one of the most impactful properties that helps govern IAV host range is the receptor-binding activity and specificity of the HA protein [15,16,17,18,19]. During IAV entry, the HA protein binds to glycoproteins and/or glycolipids on the cell surface that terminate in sialic acid (SA) [20]. Approximately 60 genes encode SA in different species [21], and these genes vary between species [22,23]. IAVs adapted to avian hosts tend to have HA proteins with receptor-binding pockets that engage alpha-2,3-linked sialic acid (SA)-containing receptors, while IAVs adapted to transmit between humans or ferrets have variations in the receptor-binding pocket that shift its form for higher-affinity binding to 2,6-linked SA-containing receptors, which are more prevalent in the human and ferret upper respiratory tract (URT) [16,24]. Swine express both forms of receptors and may serve as an intermediate host by which an avian-like or swine IAV may evolve receptor binding suitable for replication in humans [25]. In addition to differing in the attachment of terminal SA moieties, N-glycans also vary in complexity that varies by species and can modulate the receptor-binding specificities of IAVs [26]. Along with α2,3- and α2,6-linked sialylated glycans, human lungs express phosphorylated, nonsialylated glycans to which IAVs bind [27]. Overall, various host species express differing forms of glycan receptors, and HA protein adaptations in the receptor-binding pocket are often required for an IAV originating from one species to adapt to another.

As influenza-infected cells and budding IAV virions contain glycoproteins and glycolipids that have SA, IAVs have evolved an NA protein with receptor-destroying activity to limit superinfection and prevent virion–virion aggregation. Human-adapted IAVs appear to need a balance in the potency of HA receptor-binding and NA receptor-destroying activities [26,28,29,30,31], which is often referred to as HA-NA functional balance. HA stability also modulates the host range and pathogenicity of IAVs [21,32,33]. Outside the cell and host, IAVs can become inactivated after exposure to extracellular acid. Different strains vary in the pH of virion inactivation [34]. During virus entry, the HA protein is triggered to undergo irreversible structural changes that cause fusion between the viral envelope and endosomal membrane [35,36]. The pH of HA protein activation varies between different strains, and HA stability (the focus of this review) has been shown to help regulate IAV host range, infectivity, pathogenicity, and transmissibility [32,33,37].

In addition to the functional properties of the HA and NA surface antigens, internal viral genes and host genes play important roles in the biological phenotypes of IAVs [38,39,40,41,42]. After endocytosis, membrane fusion, and uncoating, the IAV genes and RNPs are transported to the nucleus [43]. Host restriction factors, including those expressed in mammalian cells, can block transit of the viral genome and RNPs into the nucleus [44]. During cytoplasmic transit, viral RNA products can be sensed by the retinoic acid-inducible gene I (RIG-I) protein, leading to a cascade of molecular interactions that lead to expression of type I and type III interferons [45], which has an antiviral effect. Entering vRNPs from avian IAVs are in general more susceptible to inactivation by RIG-I than vRNPs from mammalian IAVs, and a resistance to these antiviral effects in mammals has been mapped, in part, to the avian PB2 residue E627 being mutated to a lysine [46]. PB2-E627K and other polymerase mutations have been shown to promote adaptation of avian IAVs to mammals [47,48]. Host factors such as ANP32A [49] and others also modulate the polymerase activities of IAVs in various species, thereby contributing to host range [21]. Mutations in the M gene have also been shown to contribute to adaptation of IAVs to humans [50].

Overall, adaptation of IAVs to ferrets and humans requires a combination of viral genetic changes that are necessary for robust virus growth in an infected donor host, retention of virus infectivity while transiting between hosts, and the initiation of infection in a recipient host. These include, but are not limited to, PB2 variations (627K, 590S-591R, and 701N), α2,6-linked SA receptor-binding specificity, HA stabilization, and evasion of host restriction factors [21]. While these traits are necessary for adaptation to humans and ferrets, individually, the acquisition of isolated traits is generally not sufficient for complete adaptation of IAVs to humans or ferrets. For example, in two separate experimental adaptations of H5N1 viruses to ferrets to become airborne transmissible, all of the following changes were needed: the PB2-627K variation, two mutations that switched receptor-binding specificity to preferential α2,6-binding, a deletion of a glycosylation site that further enhanced receptor binding, and a mutation that decreased the HA activation pH to 5.4 or lower [51,52,53]. No single variation was sufficient on its own for an avian H5N1 virus to become humanized. Thus, while the focus of this review is the importance of HA stability on host range, transmissibility, and pathogenicity, it should be noted that HA stability is one of several traits that, in conjunction, modulate the biology of IAVs.

## 3. Molecular Basis of HA Stability

The HA surface glycoprotein is a type I integral membrane protein and a structural Class I viral fusion protein [35]. Uncleaved HA0 precursor protein folds as trimers in the endoplasmic reticulum (ER) in the presence of chaperones [54]. N-linked glycosylation occurs in the ER and contributes to HA protein folding [55]. During trafficking through the trans-Golgi network to the cell surface, N-linked glycosylation sites undergo maturation and unpaired cysteine residues in the transmembrane (TM) and cytoplasmic tail (CT) regions are acylated [56,57,58]. Uncleaved HA0 is not triggered to undergo structural changes by low pH and is incapable of causing membrane fusion. Instead, cleavage of HA0 into an HA1/HA2 complex is needed to prime the HA protein into a fusion-capable form [59]. The cleavage sequences, and complementary host proteases needed for processing, differ between low pathogenicity avian influenza (LPAI) and highly pathogenic avian influenza (HPAI) strains [60]. HPAI viruses have a polybasic cleavage site (R-X-R/K-R) that is cleaved intracellularly in the trans-Golgi network by subtilisin-like enzymes including furin and PC6 [61,62,63]. This is needed for systemic spread of HPAI viruses and contributes to their enhanced pathogenicity. The HA0 proteins of LPAI viruses and human IAVs are cleaved outside the cell by soluble trypsin-like proteases such as tryptase Clara [64], mini-plasmin [65], and ectopic anionic trypsin I [66]. Cell-associated serine proteases may also cleave HA0 including TMPRSS2, human airway trypsin-like protease [67], and TMPRSS4 [68]. Virions containing uncleaved HA0 proteins are protected from inactivation by a temporary reduction in pH, while those having cleaved HA1/HA2 complexes are susceptible to inactivation. Moreover, the trans-Golgi network is mildly acidic such that coexpression of the M2 protein is needed to neutralize the secretory pathway during trafficking of cleaved HA1/HA2 proteins from HPAI viruses [59].

**Figure 1:**
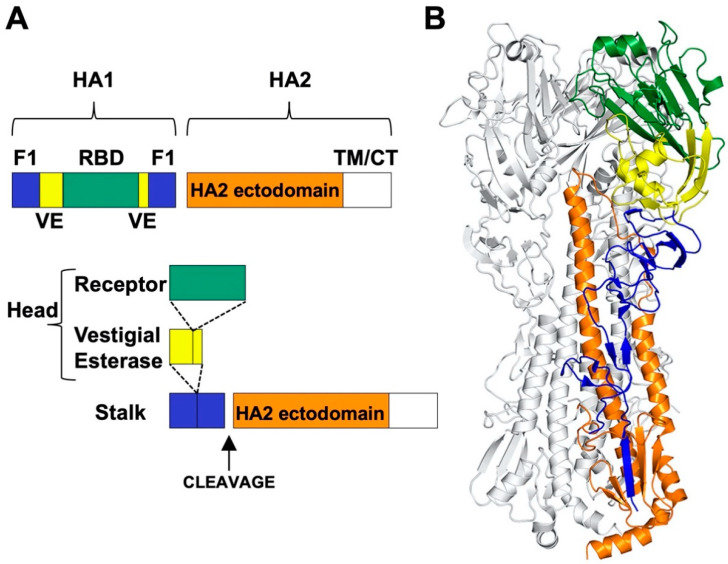
Domain arrangement and prefusion structure of the IAV HA protein. (**A**) Domain structure of the IAV HA protein. Domains in HA1 include fusion (F1, blue), vestigial esterase (VE, yellow), and receptor-binding domain (RBD, green). Domains in HA2 include the HA2 ectodomain (orange), transmembrane region (TM, white), and cytoplasmic tail (CT, white). The HA head includes the receptor-binding (green) and vestigial esterase (yellow) subdomains. The stalk (a.k.a. stem) contains the HA1 fusion domains (blue) and the HA2 ectodomain (orange). The domain insertion schematic was adapted from the influenza C HEF structure [81]. (**B**) Structure of an HA trimer in its prefusion conformation. One protomer is colored based on the conventions established in panel A, and the other protomers are colored gray. Structures were generated using MacPYMOL using A/California/4/2009 (H1N1) protein data bank structure 3UBE [82].

**Figure 2:**
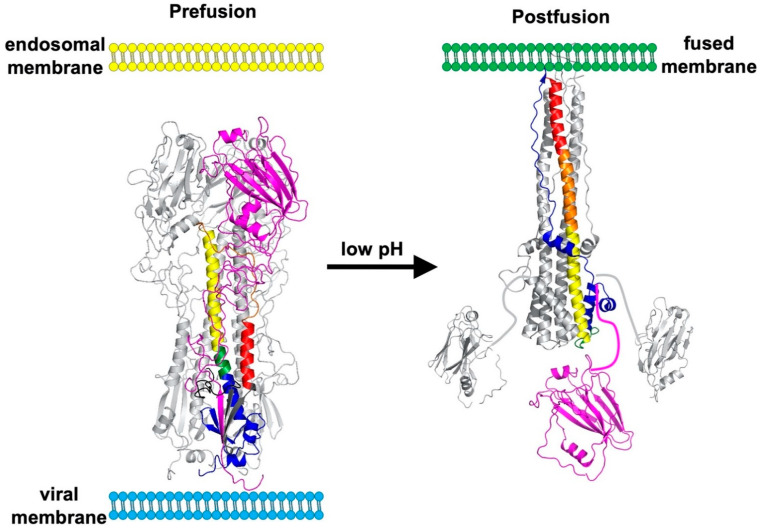
Prefusion and postfusion structures of the HA ectodomain. In the prefusion structure (**left**), the stalk domain is proximal to the viral membrane (blue lipids), and the head domain is oriented toward the endosomal membrane (yellow lipids). Two protomers are colored gray, while the third is colored as follows: HA1 (purple), fusion peptide (black), helix A (red), loop B (orange), helix C (yellow), C–D hinge (green), membrane-proximal residues (blue). After activation by low pH (**right**), the HA protein undergoes irreversible structural changes in which the head domains dissociate from the stalk and HA2 undergoes extensive refolding, helping drive fusion of the two membranes (green lipids). HA2 conformation changes include loop B continuing the central triple-stranded coil formed by helix C, the C–D hinge breaking the central helix, and the membrane-proximal residues forming a buttress antiparallel to the central coiled coil. This topology creates a hairpin structure. Structures were generated using MacPYMOL using X31 (H3N2) HA protein data bank structures 1HGF and 1QU1 [73,101].

**Figure 3:**
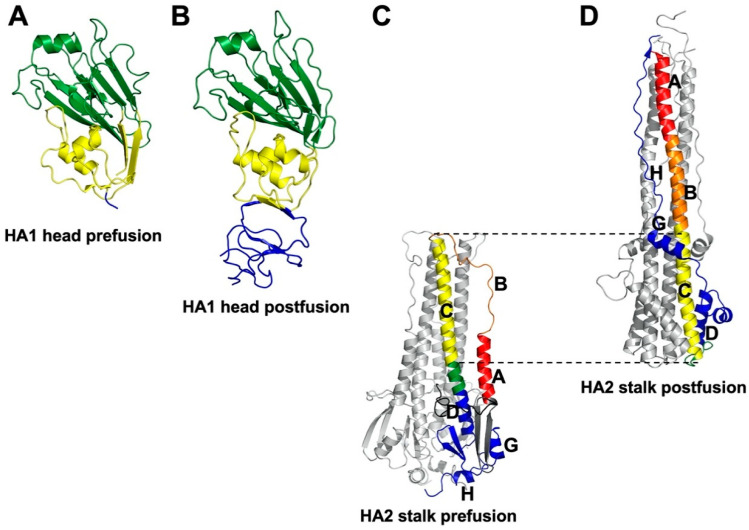
Prefusion and postfusion structures of the HA protein head and stalk. Secondary and tertiary structure is similar in prefusion (**A**) and postfusion (**B**) forms of the head. In panels A and B, the receptor-binding subdomain is colored green, the vestigial esterase subdomain is colored yellow, and portions of HA1 residues in the stalk are colored blue. HA2 residues in the stalk undergo extensive changes in secondary and tertiary structure in prefusion (**C**) and postfusion (**D**) forms. In panels C and D, the following color conventions are used: fusion peptide (black), helix A (red), loop B (orange), helix C (yellow), C–D hinge (green), membrane-proximal residues (blue). Horizontal dotted lines show how helix C is positioned similarly in panels C and D, while other regions such as loop B, the C–D hinge, and the membrane-proximal residues undergo substantial structural changes. The labeling of secondary structure elements in HA is patterned after the X31 low-pH structure [72]. Structures were generated using MacPYMOL using A/CA/04/09 (H1N1) in panel A and X31 (H3N2) in panels B–D. Protein data bank structures are 3MLH, 2VIR, 1HGF, and 1QU1 [73,77,78,101].

The HA protein contains a more-highly conserved membrane-proximal domain, called the stalk or stem, and a membrane-distal domain, called the head, which contains the receptor-binding domain and most epitopes that undergo seasonal drift (Figure 1) [36]. High-resolution, X-ray crystal structures have been obtained for several structural forms of the HA trimer ectodomain (Figure 2) including uncleaved HA0 [69], cleaved prefusion HA1/HA2 [70], an early intermediate [71], and the postfusion hairpin structure [72,73]. The early intermediate conformation has only minor changes compared to the prefusion structure and these include an outward shift in the HA2 stalk B loop and a complementary (albeit minor) deformation in the receptor-binding head [71]. HA protein structural changes are irreversible [74]. The structural changes are triggered biologically by low pH but can also be triggered experimentally by supraphysiological temperature, or mild denaturant [75,76]. The head domain can be expressed by itself and maintain its conformation at neutral and low pH (Figure 3A,B) [77,78]. The stalk domain, which contains the N- and C-terminal regions of HA1 and the ectodomain portions of HA2, undergoes a dramatic structural rearrangement after acid-induced activation (Figure 3C,D) [70,72]. Membrane fusion occurs by a spring-loaded mechanism in which a hairpin structure forms and juxtaposes membrane-interacting fusion peptide and transmembrane (TM) domains, membrane disruption occurs, and multiple trimers open and enlarge a fusion pore sufficiently to allow passage of viral RNPs [79,80].

Mutations that alter HA stability, often measured as the pH of HA activation or the pH of virion inactivation, are located throughout the primary structure in regions that undergo changes in secondary and tertiary structure during protein refolding [83,84,85,86,87,88]. The ubiquitous nature of HA stability-regulating residues was first noted when a large panel of amantadine-selected mutants were mapped on the prefusion and postfusion structures [70,72,83]. Subsequent reviews include additional mutations and the same conclusion: HA stability is regulated by many residues located throughout the trimer and the property should be monitored phenotypically, not genotypically [32,33,37]. A few mutations have similar effects across subtypes [89,90]; however, most stability altering mutations are strain specific. It is unlikely that HA stability can be inferred from calculations using protein structures as the structures of fusion mutants usually contain identical backbones with substitute residues forming alternate noncovalent bonds [91,92,93,94,95]. Creation or destruction of salt bridges, especially between structural domains, usually has an impact on HA stability and could be used in predictive algorithms [96]. However, HA stability can be modulated by NA activity [97,98,99] and the matrix gene segment [100], further complicating any attempt to predict HA stability based on its gene sequence. Numerous mutations that alter HA stability are described in detail in the subtype-specific sections below.

The HA proteins from IAVs form two distinct structural groups [102]. Group 1 HA proteins include H1, H2, H5, H6, H8, H9, H11, H12, H13, H16, H17, and H18. Group 2 HA proteins include H3, H4, H7, H10, H14, and H15. The first HA structure was of the HA protein from virus X-31, which contains the HA protein from A/Aichi/68 (H3N2) [70]. Different subtypes have insertions and deletions, so the structural field has used H3 numbering for all subtypes. This assists in contemplation of critical residues in the receptor-binding pocket yet is a source of confusion for those outside of the structural field. A universal numbering scheme for IAVs across HA subtypes has been described [103], and an HA-subtype numbering conversion program has been released as a beta version on Fludb.org [104]. In this review, H3 numbering is used.

Membrane fusion mediated by the IAV HA protein is a field onto itself [105]. A variety of assays have been developed to measure HA conformational changes, membrane fusion, and virion inactivation [106]. Most assays measure the bulk properties of virions or HA-infected/HA-expressing cells; however, advanced techniques have been developed to measure single-particle fusion kinetics and activation pH values [107,108]. The most common technique for measuring HA conformational changes is to expose aliquots of HA protein to various pH buffers and treat with trypsin as the prefusion form is protected from digestion. Conformation-specific monoclonal antibodies may also be used if available but are strain-specific. The pH of membrane fusion has been measured as the pH at which (a) virions cause membrane disruption of red blood cells (RBCs) in a hemolysis assay, (b) virions fuse to liposomes, or (c) cell-expressed HA protein causes syncytia formation or cell-to-cell fusion by reporter assays. The pH of virion inactivation can be measured by exposing aliquots of virus to solutions of varying pH and measuring residual infectivity by standard assays (e.g., TCID_50_ or plaque assay) or residual receptor-binding activity by the hemagglutination assay (HA assay). For HA mutants in a similar genetic background, HA stability has been shown to correlate with thermal stability [75]. Therefore, the thermal stability of the HA protein has been probed by heating aliquots of virus at supraphysiological temperatures (often 50–60 °C) and measuring residual infectivity or HA activity. However, the relationship between HA activation pH and virion thermostability has not been properly addressed for differing IAV strains, making it difficult to recommend thermal stability assays as a primary means to probe HA stability.

## 4. Trends in HA Stability for Viruses Isolated from Different Species

HA stability values have been measured for many IAV strains, and many of these studies are described starting in Section 6. Several studies that measure HA stabilities across subtypes are worth noting. The pH and temperature of IAV virus inactivation has been measured for 34 IAVs of varying subtype isolated from humans, swine, and avian hosts [34]. An analogous study has measured the pH of membrane fusion induced by the HA proteins of 25 IAVs of varying subtype [109]. The pH of hemolysis has been measured for a wide variety of avian IAV isolates [110]. The environmental persistence of varying avian, swine, and human IAVs in solution and on surfaces have also been compared in several large-scale studies [111,112,113].

Overall, and described in detail below, IAVs range in HA activation pH from approximately 4.8 to 6.2. Some cultured cells prefer low or high HA stability, suggesting that this molecular property may play a role in tropism. In general, avian and swine IAVs have relatively broad ranges of HA stability, although some individual clades appear to prefer either relatively high or low HA stability. Optimal replication in mice often occurs when the HA protein has an activation pH of approximately 5.5, with higher and lower HA stability resulting in attenuation. Human- and ferret-adapted IAVs seem to require an HA activation of approximately pH 5.5 or below for efficient airborne transmissibility. Therefore, risk assessment algorithms that survey emerging viruses for pandemic and pathogenic potential in humans should explicitly consider HA stability in addition to receptor-binding specificity.

## 5. Animal Models

IAVs circulate in a reservoir of wild aquatic birds and have spread to many other species including aquatic mammals (e.g., seals and whales), terrestrial avians (e.g., wild turkeys, quail, and ostrich), domestic birds and livestock (e.g., chickens, turkeys, swine), pets (e.g., horses, dogs, and cats), and humans [1,2]. IAVs isolated from wild aquatic birds include H1-H16 and N1-N9 subtypes, and most of these subtypes have been isolated from chickens [114,115]. H1N1, H1N2, and H3N2 subtypes are endemic in swine [12]. Known human pandemic/seasonal viruses include H1N1 (1918, 1977, and 2009), H2N2 (1957), and H3N2 (1968) [2,116,117]. H1 and H13 viruses have been isolated from whales [118], and H1-H4, H7, and H10 viruses have been recovered from seals [119,120]. H5, H7, H9, and H10 viruses have been isolated from ostriches [121], while H3N8 and H7N7 have infected horses [122]. Dogs have become infected with H5N1, H3N2, and H3N8 [123], while cats have been infected with H1N1, H3N2, and H5N1 [124,125].

A variety of animal models have been used to study IAV infection [126,127,128]. Chickens are commonly used to measure pathogenicity of avian IAVs, and other studies have investigated replication, pathogenesis, and transmissibility in waterfowl and domestic birds [129,130,131]. Mice are a convenient small-animal model, and numerous studies have dissected the molecular determinants of IAV adaptation to and pathogenesis in mice [132]. Different strains of inbred mice vary in their susceptibilities to IAV infection with DBA/2 mice being relatively susceptible and BALB/c and C57/Bl6 mice being relatively resistant [133]. Swine are considered an intermediate host that can facilitate the acquisition of human pandemic potential by avian-origin viruses. Therefore, swine models have been developed to study IAV infection, adaptation, transmission, and immunity [134]. Guinea pigs are an attractive small-animal species to study IAV transmission, and their small size is convenient for studying the importance of environmental factors (e.g., temperature and humidity) [135]. The ferret is often considered as the gold-standard animal model for human-like infection in pathogenesis and transmission studies [136,137,138]. In ferrets, transmitting infectious IAV particles appear to arise from the URT [139,140], and the soft palate may also contribute to adaptation [141]. Avian-like IAV H4N6 viruses isolated from seal have also been studied [119].

## 6. H1N1

### 6.1. Seasonal H1N1

The HA protein from a 1918 pandemic virus has an HA activation pH of 5.6, while a constructed 1918-like avian HA protein has an activation pH of 5.3 [142]. **A/Puerto Rico/8/34 (PR8) (H1N1)** has been reported to have a virion inactivation pH of 5.4 [34] and an HA activation pH of 5.0–5.1 [109]. To enhance influenza virus vaccine seed stock growth in a World Health Organization (WHO)-recommended cell line, PR8 was passaged 11 times in Vero cells [143]. Mutation HA2-N117D was primarily responsible for increased growth in Vero cells. Using a cell–cell fusion reporter gene assay, PR8 had an HA activation pH of 5.2 while the HA2-N117D adaptation increased this value to 5.4 (Table 1). HA2-N117D was introduced into pandemic strain **A/California/4/2009 (H1N1)** and seasonal strains **A/Kawasaki/173/01 (H1N1)** and **A/Kawasaki/UTK-4/09 (H1N1)**. The functionally equivalent H3-subtype mutation HA2-N116D was introduced into **A/Yokohama/2013/2003 (H3N2)**. PR8/H1N1 or PR8/H3N2 6:2 reassortant viruses were generated, and all viruses grew to higher titers in Vero cells but not in MDCK cells. Using dextran-conjugated fluorescent dyes as pH sensors, Vero cells were shown to have a higher endosomal pH than MDCK cells. Overall, an HA activation pH of 5.2 was shown to be sufficient for growth of H1N1 viruses in MDCK cells but a value of 5.4 generated higher yields in Vero cells. This is most likely due to the endosomal pH of Vero cells being higher than that of MDCK cells such that viruses with relatively low activation pH values (e.g., pH 5.2) are not as efficiently triggered for entry in Vero cells [143].

A highly virulent variant of **PR8** (hvPR8) was found to contain HA mutations [144]. These mutations altered receptor-binding and HA activation properties. Using a cell–cell luciferase reporter assay, the lower-virulent strain had an HA activation pH of 5.3 and combined HA1-P78L and HA2-H25Q increased the HA activation pH of hvPR8 to 5.5 (Table 2). hvPR8 also contained mutations in PB2 that affected pathogenicity. In summary, in the background of PR8, an HA activation pH of 5.5 was shown to contribute to higher pathogenicity in mice compared to pH 5.3.

**A/FM/1/47-MA (H1N1)** is a mouse-adapted variant that has increased virulence due to HA, M1, and perhaps other mutations [147]. While A/FM/1/47 WT virus caused hemolysis at approximately pH 5.7, the mutant virus containing an HA2-W47G mutation decreased the pH of hemolysis to approximately pH 5.5 (Table 2). Thus, an activation pH of 5.5 was associated with enhanced virulence of A/FM/147 (H1N1) in mice compared to pH 5.7. **A/WSN/33 (H1N1)** WT was generated by reverse genetics along with two single and one double mutant containing HA2-T64H and/or HA2-V66H [148]. In a cell–cell luciferase assay, WT had an HA activation pH of 5.4, while the double mutant decreased the HA activation pH to 5.2. The double mutant attenuated virus replication and pathogenicity in mice yet yielded higher titers at later timepoints (days 8 and 10 p.i.) in the lungs and brains (Table 2). In summary, compared to WSN WT, which has an HA activation pH of 5.4, stabilizing mutations that decreased the HA activation pH to 5.2 caused attenuation.

### 6.2. Pandemic pH1N1

pH1N1 emerged from swine in Mexico in early 2009 [149,150,151]. The segments PB2, PB1, PA, NP, and NS were from a triple reassortant H3N2 swine virus (TRsw) from the North American lineage, the HA segment was from the classical swine H1N1 lineage (Csw) that circulated in North American swine since the 1918 pandemic, and the NA and MP had segments were related to the avian-like Eurasian swine lineage (EAsw). In syncytia assays, **pre-2009** **swine viruses containing Csw HA genes** have been found to range in HA activation pH from 5.5 to 5.8 [152]. By various assays, human pandemic isolates **A/California/04/2009 (H1N1), A/Tennessee/1-560/2009 (H1N1)**, and **A/England/195/2009 (H1N1)** have HA activation pH and virion inactivation pH values that both are approximately 5.5–5.6 [109,152,153]. Thus, HA activation pH values for early human pH1N1 isolates are at the lower end of the range of precursor Csw viruses.

The HA ectodomains of 2009 pH1N1 isolates are less stable compared to those from other subtypes [109], and ectodomain trimers can reversibly form monomers [155,156], which was unexpected compared to other subtypes. Furthermore, this ectodomain lability can expose an inter-monomer epitope [157]. Within less than one year, the variant HA2-E47K became dominant in circulating human pH1N1 [158,159]. **A/California/7/2009 (H1N1)** virions are inactivated upon exposure to pH 5.5 media, and the HA2-E47K mutation stabilizes the HA protein such that virion inactivation pH is decreased to pH 5.3 [146]. The seasonal isolate **A/Brisbane/10/2010 (H1N1)** has an HA activation pH of 5.3, and the reverse mutation (i.e., HA2-K47E) increases the HA activation pH to 5.5 [146]. Other pH1N1 variations that have been maintained in the human population, which are not likely due to antigenic drift and are located at positions that may alter the stability of the trimer, are HA1-D104N, HA1-S206T, HA1-A259T, HA1-K285E, HA2-S124N, and HA2-E172K [160]. In general, as pH1N1 has evolved in humans, the HA protein has become more stable [161]. While 2009 pH1N1 isolates have HA activation pH values of approximately 5.5, isolates from 2010 to 2012 have been shown to range from pH 5.2–5.4 [152]. Therefore, the pH1N1 HA activation pH was approximately 5.5 at the start of the 2009 pandemic and decreased after adaptation to humans.

**A/Hamburg/04/2009** was passaged 6 times in A549 human lung epithelial cells, yielding a variant with 100-fold higher replication [145]. The adaptive mutations causing increased virus growth were HA1-D130E, near the receptor-binding site, and HA2-I91L, in the stalk. Cell–cell dye transfer assays using transfected plasmids showed the HA2-I91L mutation increased the HA activation pH to approximately 5.9 compared to WT, which had a value of approximately 5.7. After 48 h infection in mice, the HA1-D130E receptor-binding mutation increased viral loads in the lungs by approximately 10-fold, while the HA2-I91L destabilizing mutation increased virus in the lungs by approximately 2-fold. Thus, an increase in HA activation pH from 5.7 to 5.9 was shown to increase the replication of A/Hamburg/04/2009 100-fold in A549 cells and by a small, but substantial, amount in mouse lungs. Follow-up studies are needed to determine if the destabilizing mutation affects weight loss and survival in mice.

A pH1N1 isolate from a fatal case contained amino-acid variations PB2-A221T, PA-D529N, and HA1-S110L [162]. HA1 residue 110 is located at the interface of the head and the stalk domains, and an HA1-H110Y mutation has been shown to stabilize the H5 HA protein [53]. Recombinant viruses containing these mutations were created in the background of **A/California/04/2009 (H1N1)**. Both WT and HA1-S110L had virus inactivation pH values of approximately 5.5 and were equally susceptible to chloroquine-induced attenuation in NIH 3T3 cells. Both viruses had similar multistep replication kinetics in A549 cells. The HA1-S110L mutant induced Mx and ISG56 antiviral responses in A549 cells at a lower level and had a higher LD50 in mice. Overall, the PA-D529N variation was found to be predominantly responsible for increased virulence, and the HA mutation apparently modulated host responses by an unknown molecular mechanism that is not HA stability.

The six internal genes of the cold-adapted, **live-attenuated vaccine strain A/Ann Arbor/6/60 (AA ca, H2N2)** were used to generate reassortants containing the HA and NA genes of **A/California/7/2009 (H1N1)** and **A/Brisbane/10/2010 (H1N1)** [146]. Cal/09 HA-WT and HA2-E47K had virus inactivation pH values of 5.5 and 5.3, respectively. Bris/10 HA-WT and HA2-K47E had virus inactivation pH values of 5.3 and 5.5, respectively. The mutations did not affect multistep replication kinetics in MDCK cells. However, in Vero cells the viruses containing HA2-E47 in HA2, and an HA activation pH of 5.5, had accelerated replication kinetics and grew to higher titers than the viruses containing HA2-K47 (pH 5.3) (Table 1). The stabilized HA2-K47 variants also had slower inactivation kinetics at 4 and 26 °C. In ferrets, the stabilizing HA2-E47K mutation decreased the FID_50_ value of A/California/7/2009 (H1N1) from 17.8 to 3.2 PFU, while the destabilizing HA2-K47E mutation increased the FID_50_ value of A/Brisbane/10/2010 (H1N1) from 4.4 to 23 PFU (Table 2). Overall, a decrease in virion inactivation pH from 5.5 to 5.3 was associated with reduced growth in Vero cells, increased thermostability, and a higher infectivity in ferrets for the 6:2 vaccine recombinants. While a relatively unstable HA protein may have contributed to the reduced vaccine efficacy of Cal/09 live-attenuated vaccines in the period 2013–2014, a follow-up vaccine candidate **A/Bolivia/559/2013 (H1N1)** had a sufficiently stable HA protein yet was poorly immunogenic. Therefore, suboptimal HA stability was most likely not the dominant factor in reduced vaccine efficacy of live-attenuated pH1N1 viruses in the 2013–2014 and 2015–2016 seasons [163].

**A/Tennessee/1-560/2009 (H1N1)** was shown to have an HA activation pH of 5.5 by syncytium formation assay, while a mutant virus containing HA1-Y17H had an HA protein activated at pH 6.0 [152]. The two viruses were shown to have similar multistep replication kinetics in the following cell lines inoculated with 0.01 PFU/cell: MDCK, A549, normal human bronchial epithelial (NHBE), and LA-4 mouse lung adenoma cells (Table 3) [152,154]. When inoculated at a high MOI of 3 PFU/cell, the HA1-Y17H mutant had accelerated replication kinetics in MDCK and A549 cells. Replication of HA1-Y17H was attenuated in murine nasal and tracheal epithelial cells (mNECs and mTECs). HA1-Y17H enabled productive replication in RAW 264.7 murine macrophage cells, unlike WT virus [154]. Similarly, H5N1 and seasonal H1N1 viruses with HA activation pH values of approximately 5.9 overcome an early block in the replication cycle of macrophages via a relatively unstable HA protein [164,165,166], presumably by entry occurring in early versus late endosomes. Overall, for A/Tennessee/1-560/2009 (H1N1), the destabilizing HA1-Y17H mutation was shown to enable productive infection in macrophages, allow accelerated early replication in MDCK, A549, and dendritic cells when inoculated at a high MOI, but reduce replication in murine airway epithelium cultures when inoculated at a low MOI. Thus, the change in HA stability substantially altered the tropism of the virus in cultured cells.

The effects of HA stability mutations on pH1N1 replication in cell culture have been found to be similar for **A/England/195/2009 (H1N1)**, which has an HA activation pH of 5.5 and a virion inactivation pH of 5.45 [153]. In the background of this virus, HA1-E31K is stabilizing (activation pH 5.3; inactivation pH 5.15) and HA1-A19T (5.8; 5.55) and HA1-Y17H (5.9; 5.75) are destabilizing. When inoculated at a high MOI, the destabilized mutants HA1-A19T and HA1-Y17H produced a higher signal in virus-driven replicon assays and grew to higher titers in MDCK and A549 cells (Table 3). This advantage by viruses containing a destabilized HA protein pinpointed to entry in early endosomes [153]. In primary HAE cells inoculated at a low MOI, a reverse effect was seen where HA1-A19T (activation pH 5.8) was attenuated compared to WT (activation pH 5.5) and HA1-Y17H (activation pH 5.9) was further attenuated (Table 3). Thus, the observed effects of changes in HA stability on pH1N1 growth in cultured cells were similar in two laboratories using two different 2009 pH1N1 isolates (Table 3) [152,153,154].

In BALB/c mice inoculated with 200,000 PFU of **A/England/195/2009 (H1N1)**, WT virus (HA activation pH 5.5) grew to an average lung titer up to 10-fold higher than HA1-E31K (pH 5.3), HA1-A19T (pH 5.8), and HA1-Y17H (pH 5.9). Moreover, WT caused approximately 9% loss in body weight, while HA1-E31K and HA1-A19T caused approximately 5% and HA1-Y17H did not cause a loss in body weight. Overall, the pH1N1 virus with an HA activation pH of 5.5 replicated to the highest level and was the most pathogenic in mice; viruses with greater or less HA stability were attenuated (Table 2).

BALB/c mice are relatively resistant to infection by non-adapted pH1N1 viruses [133]. Therefore, the impact of HA stability of **A/Tennessee/1-560/2009 (H1N1)** was investigated using DBA/2J mice (Table 2) [152,154], which are more susceptible to IAV infection. In this context, the destabilizing HA1-Y17H mutation (HA activation and virion inactivation pH of 6.0) increased the MID_50_ from 4.7 to 48 PFU compared to WT (pH 5.5) and increased the MLD_50_ from 11,000 to >375,000 PFU [154]. The stabilizing HA2-R106K mutation (HA activation pH 5.3; virion inactivation pH 5.4 [152]) decreased the MID_50_ from 4.7 to 0.68 PFU compared to WT but increased the MLD_50_ from 11,000 to 20,100 PFU. Thus, for mice infected with A/England/195/2009 or A/Tennessee/1-560/2009, an HA activation pH and virion inactivation pH of approximately 5.5 was associated with greatest pathogenicity, and an increase or decrease in HA stability is attenuating (Table 2) [152,153,154]. Attenuation in mice via HA destabilization has been associated, in part, with (a) earlier entry into dendritic cells, (b) increased type I interferon (IFN) responses, (c) more extensive changes in host gene expression, and (d) reduced virus replication, inflammation, and pathology in the lungs [152,154]. In this way, HA stabilization appears to decrease the immunological stealthiness of pH1N1 [167].

Using reassortants of **A/Bretagne/7608/2009 (H1N1)**, **A/WSN/1933 (H1N1)**, and **A/Paris/2590/2009 (H1N1)**, environmental persistence has been mapped to the stability and expression level of the HA protein in addition to calcium-binding sites in the NA protein [168]. Thus, HA stabilization has been shown to enhance the longevity of pH1N1 virions.

Extracellular lung pH in DBA/2J mice was measured to range from pH 7.0 to 7.4 before and during infection with **A/Tennessee/1-560/2009 (H1N1)** [154]. While the HA1-Y17H mutant is inactivated at an accelerated rate compared to WT when exposed to pH 6.4 media, the environmental persistence of HA1-Y17H is like that of WT at pH 7.0 [154]. Therefore, HA1-Y17H is most likely not attenuated in the lungs of DBA/2J mice due to extracellular inactivation.

**A/Tennessee/1-560/2009 (H1N1)** is an early pandemic virus and has all the required properties for pandemic potential. To test the hypothesis that a stable HA protein is required for airborne transmissibility in ferrets, transmission studies were performed using A/Tennessee/1-560/2009 WT (pH 5.5) and HA1-Y17H (pH 6.0) (Table 4). Peak nasal titers in the HA1-Y17H group were delayed 3 days and reduced 100-fold compared to the WT group [152]. Contact transmission by HA1-Y17H was delayed 2–4 days, and in some cases yielded subpopulations of HA-stabilizing variant HA2-R106K and the revertant HA1-H17Y. WT transmitted by the airborne route with 100% efficiency (4/4). The HA1-Y17H group airborne transmitted in 1/4 cases, and the recovered virus had a both HA2-R106K and HA1-H17Y mutations, which lowered the HA activation pH from 6.0 to 5.3. These results showed that a human pandemic-capable virus, A/Tennessee/1-560/2009, was incapable of airborne transmission in ferrets without a stabilized HA, despite having all other molecular traits needed for human adaptation including a preference for binding alpha-2,6-linked SA and mammalian-capable polymerase complex. The fact that this regain-of-function experiment in ferrets recapitulated the phenotypic evolution of the pH1N1 HA protein as it adapted to humans supports the use of ferret airborne transmission studies as a marker for human pandemic potential.

**A/England/195/2009 (H1N1)** has also been used as a model to investigate the impact of HA stability on the emission from and transmissibility between ferrets [169]. While similar amounts of infectious virus were recovered from the nasal washes of ferrets infected with HA1-E31K (HA activation pH 5.3; virus inactivation pH 5.15) and HA1-Y17H (activation pH 5.9; inactivation pH 5.75), the stabilized HA1-E31K variant was recovered from emitted airborne droplets at a substantially higher level (184 plaques compared to 23) [153,169]. Furthermore, inoculated ferrets and air-emitted virus from the HA1-Y17H group contained the stabilizing variants HA1-Y17H, HA2-V55I, HA2-E47K, and HA1-V29I (Table 5) [169]. After nebulization of a 40:60% mixture of HA1-E31K and HA1-Y17H viruses, the stabilized HA1-E31K variant became enriched to of a level of approximately 65–79% abundance. In summary, HA stabilization has been shown to promote airborne transmission of pH1N1 in ferrets by increasing virus survival after exhalation.

**A/California/07/2009 (H1N1)** WT has been shown to retain infectivity for over 1 h in both fine aerosols and stationary droplets over relative humidity ranging from 23 to 98% [170]. Environmental persistence after 2–16 h of IAVs in suspended aerosols and stationary droplets under a range of RH conditions has been shown to depend on virus strain and host origin [171]. For example, in the presence of human airway surface liquid, **A/Perth/16/16/09 (H3N2)** and **B/Texas/02/2013** are resistant to RH-dependent inactivation, while the decay **of A/California/07/2009 (H1N1)** over 16 h was accelerated more at RH values of 23% and 98% than at 43% and 75%. Overall, the persistence of influenza virus in aerosolized particles appears to be influenced by droplet evaporation and its impact on droplet physics [172], both of which may contribute to inactivation of pH1N1 after emission from infected hosts [169].

pH1N1 emerged from swine [149,150,151] and swine can serve as an intermediate host for avian-to-human adaptation of IAVs. Therefore, the importance of HA stability for replication in swine has been investigated. In swine, **A/Tennessee/1-560/2009** WT (pH 5.5) and HA2-R106K (pH 5.3) were shown to grow to similar levels, transmit with 100% efficiency to co-housed swine, and transmit with 100% efficiency to separately caged ferrets with no notable changes in HA sequence or stability (Table 6) [173]. Thus, a virus with a relatively stable HA activation pH of 5.3 is fully capable of robust growth and contact transmission in swine, at least in the context of pH1N1. In contrast, HA1-Y17H (pH 6.0) has delayed growth in inoculated and contact swine, and the virus requires stabilizing mutations to airborne transmit from swine to ferrets. A stabilizing mutation arising during swine-to-swine transmission was HA2-K153E (pH 5.3), and one arising during swine-to-ferret transmission was HA2-V55I (pH 5.2). These experiments show that an HA activation pH of 6.0 is too high for efficient replication of a pH1N1 virus in swine or airborne transmission to ferrets. As swine support efficient pH1N1 replication and transmission by a virus containing a stabilized HA protein (i.e., HA2-R106K, pH 5.3), swine may be well suited as an intermediate host for humanization of both HA stability and receptor-binding specificity [173].

### 6.3. Swine and Avian H1

**Swine H1N1v and H1N2v variants** have infected humans, and risk assessments studies have been performed that have measured their HA stabilities [174]. **A/Texas/14/2008 (H1N1v), A/Ohio/09/2015 (H1N1v),** and **A/Wisconsin/71/2016 (H1N2v)** were shown by syncytia assay to have HA activation pH values of 5.7. **A/Ohio/02/2007 (H1N1v), A/Iowa/39/2015 (H1N1v),** and **A/Minnesota/19/2011 (H1N2v)** have HA activation pH values of approximately 5.6. **A/New Jersey/08/1976 (H1N1)** and **A/Minnesota/45/2016 (H1N2v)** have activation pH values of approximately 5.5. In Calu-3 human bronchial epithelial cells, OH/09, IA/39, MN/45, MN/19, and WI/71 achieve similar peak titers at 33 and 37 °C by 72 h. In BALB/c mice, WI/71 (pH 5.7) grows to a level 10-fold higher after 3d of infection, and OH/09 (pH 5.7) causes approximately 80% mortality while all mice infected with the other variants survive. Thus, the variants with a higher activation pH had superior growth and pathogenicity properties in mice. Ferrets were infected with three variants: MN/45 (pH 5.5), MN/19 (pH 5.6), and WI/71 (pH 5.7). All three had maximum nasal wash titers in infected ferrets of approximately 6.7 log10 PFU/mL, and all three transmitted 100% (3/3) by direct contact. Out of 3 airborne recipients for each group, MN/45 transmitted to 3/3, MN/19 to 1/3, and WI/71 to 2/3. In summary, A/Minnesota/45/2016 (H1N2v, pH 5.5) had 100% airborne transmission and the other two variants (pH 5.6–5.7) had intermediate airborne transmissibility. In a follow-up study, A/Minnesota/45/2016 was shown to remain infectious longer after aerosolization than other variants that do not airborne-transmit as efficiently [175].

In approximately 1999, the gamma clade of the H1N1 Csw lineage split into two branches that include the swine gamma clade and swine viruses that later contributed the HA gene to 2009 pH1N1 (pandemic clade) [12]. **Swine gamma viruses isolated in** the period **2012–2016** have been shown to have HA activation pH values ranging from 5.5 to 5.9, while human pH1N1 and swine H1N1 of the pandemic clade isolated in the period 2009–2016 have HA activation pH values ranging from 5.0 to 5.5 [176]. The gamma-clade swine viruses with a higher HA activation pH were found to replicate to a higher level in MDCK cells than the human pH1N1 and swine pandemic-clade viruses. In ferrets, airborne transmission was only observed for gamma-clade swine viruses having a relatively stable HA protein (HA activation pH of 5.5–5.6). After inoculation of a mixture of **A/swine/Illinois/2A-1213-G15/2013 (G15)** that contained 85% HA1-N210 (pH 5.8) and 15% HA1-S210 (pH 5.5), the stabilized HA1-S210 variant outgrew the destabilized variant in the nasal washes of inoculated ferrets within 1–3 days and was 100% (3/3) transmitted by the airborne route (Table 7). Overall, a stabilized HA (pH 5.5–5.6) was shown to promote upper-respiratory tract growth in infected ferrets and be necessary for airborne transmission of swine H1N1 gamma viruses.

The HA stabilities of **EAsw viruses** isolated from swine and **closely related H1N1 viruses isolated from wild aquatic birds** have been described [177]. In MDCK cells, the EAsw viruses were less sensitive to neutralization by NH_4_Cl (a lysosomotropic agent that increases endosomal pH), having IC_50_ values ranging from 0.83 to 1.80 mM compared to 0.26–0.55 mM for the avian viruses. Additionally, the EAsw viruses had hemolysis activation pH values of 5.07–5.39 and virus inactivation pH values of 5.47–5.91. On average, the avian viruses had lower hemolysis pH values (4.96–5.20) and inactivation pH values (5.19–5.43). Another study reported EAsw viruses having HA activation pH values of pH 5.8–6.0 by syncytia formation assay, while avian H1 viruses ranged from 5.5 to 6.1 [152]. To resolve these discrepancies [152,177], larger samples of EAsw and avian H1 viruses need to be characterized using multiple HA activation assays including hemolysis and syncytia formation. In general, avian and swine IAVs may vary broadly in HA activation and virion inactivation pH with specific clades having greater or less HA stability.

Consistent with the notion that not all avian IAVs have relatively unstable HA proteins, **A/shorebird/DE/300/2009** WT has been shown to have an HA activation pH of 5.0 by hemolysis assay and a virion inactivation pH of 5.3 [178]. Adaptation to the intestinal tracts of DBA/2J and BALB/cJ mice resulted in the selection of fecal variants that have increased pathogenicity in mice. These fecal isolates acquired mutations that increased the HA activation pH to 5.2 and the virion inactivation pH to approximately 5.5. Thus, intestinal passage of sb/DE/09 resulted in a 0.2 pH unit increase in HA activation and virion inactivation pH.

In another study, **A/mallard/Netherlands/10-Nmkt/1999 (H1N1)** was passaged ten times in newborn pig trachea cells [179]. WT virus was shown by hemolysis assay to have an HA activation pH of approximately 5.2. An HA1-S133F mutation increased replication in swine cells and increased the midpoint of hemolysis to pH 5.5 [179].

Studies on H5N1 viruses [51,52,53,92,99,180,181] have suggested that HA activation pH values of avian viruses tend to be higher than those adapted to humans [32]; however, this has not been supported by subsequent studies including those on H1 viruses. While specific clades of avian and swine IAVs may trend toward relatively high or low HA stability, avian and swine IAVs appear to have broad ranges in HA stability.

## 7. H2N2 and H2N3

Relatively little is known about the impact of HA stability on H2 viruses. **A/Singapore/1/57 (H2N2)** has been reported to have a virus inactivation pH of 4.8 or lower [34]. Cell-expressed HA proteins from **A/Japan/305/1957 (H2N2)** and **A/duck/Germany/1215/1973 (H2N3)** have been shown to promote cell-to-cell fusion at pH 5.2 and 5.4–5.5, respectively [109]. Thermal inactivation at 55 °C of **seven H2N3 mallard duck isolates** has been shown to occur as a two-step process, suggesting that either two or more populations of viruses or two or more viral properties have different rates of virus inactivation at supraphysiological temperature [182]. Plaque-purified **A/Mallard/Alberta/205/1998 (H2N3) variants** formed either large or small plaques, but all had similar, linear thermal decay kinetics, unlike the biphasic parental virus sample. Compared to the parental virus A/Mallard/Alberta/205/1998, plaque-purified populations had HA1-K156E, HA1-G227R, HA2-M84V, and HA2-I133T variations, and these were suggested to increase H2N3 longevity at 55 °C. The effects of these mutations have not been addressed experimentally. Additionally, differences in plaque size that did not alter thermal stability were thought to arise from variations in the PA and PB2 polymerase genes.

## 8. H3N2

**A/Aichi/2/1968 (H3N2)** and **A/Victoria/3/1975 (H3N2)** have HA activation pH values of 5.2 and 5.0, respectively, and **A/Hong Kong/68 (H3N2)** has a virus inactivation pH of 5.1 [34,109]. **A/Bethesda/55/2015 (H3N2)** has a syncytia formation pH of 5.45 and a virion inactivation pH of 5.55 [183]. HA activation pH values measured by syncytia formation for a panel of **57 H3N2 swine IAVs isolated in** the period **2010–2011** range from 5.3 to 5.8 [173]. **Six duck, chicken, and goose H3N2 viruses isolated from 1963** to **1977** have virus inactivation pH values ranging from 5.1 to 5.4 [34].

In addition to A/chicken/FPV/Weybridge (H7N7), growth of **X-31 (H3N2)** in the presence of a high concentration of amantadine hydrochloride, which raises endosomal pH, selects variants that increase the pH of hemolysis and HA conformational changes as measured by trypsin susceptibility [83]. After passage in MDCK cells, egg-grown X-31 viruses (containing mutations to cysteine residues in the cytoplasmic tail) were found to acquire HA mutations that increase the pH of trypsin susceptibility, liposome fusion, and syncytia formation by 0.1–0.4 pH units [184]. Overall, H3N2 strains that have relatively low HA activation pH values have been shown to select for destabilizing mutations when passaged under certain mammalian cell culture conditions.

Adaptation of a 6:2 reassortant virus with the internal genes of **WSN** and the HA and NA genes from **A/Perth/16/2009 (H3N2)** in MDCK-SIAT1 cells resulted in HA1-G78D and HA1-T212I mutations [185]. These mutations increased the pH of virus inactivation from 5.5 to 5.8 [186]. The mutations also increased replication in MDCK cells but reduced airborne transmission in ferrets from 3/3 to 1/3. Noncoding regions in the NA gene contributed to the change in the pH stability of the virus. Overall, this work is significant as it extends to the H3N2 subtype a requirement of acid stability for airborne transmissibility in ferrets and identifies a role for NA non-coding regions in modulating acid stability.

Four **swine-origin H3N2 variant (H3N2v) viruses** isolated from humans in the period 2011–2016 have been shown by syncytia assay to have HA activation pH values that range from pH 5.5–5.6 [187]. **A/Ohio/13/2012** (**H3N2v**, pH 5.6) and **A/Ohio/27/2016 (H3N2v**, pH 5.5) were able to transmit by the airborne route in ferrets with 100% efficiency (3/3), while **A/Iowa/8/2011** (**H3N2v**, pH 5.6) and **A/Michigan/39/2015** (**H3N2v**, pH 5.6) did not (0/3).

**A/canine/Illinois/41915/2015 (H3N2)** has been shown to have an HA activation pH of 5.5 and a virus inactivation pH of 5.1, while **rg-A/canine/Indiana/1117-17-1/2017 (H3N2**) has HA activation and virion inactivation pH values of 5.5 and <5.2, respectively [183]. The divergence between HA activation pH and virion inactivation pH in these viruses is noteworthy, and it is presently unknown which property is more important in determining influenza virus host range and transmissibility. Both canine viruses were shown to bind preferentially to α2,3-linked sialic acid (SA) receptors like other Eurasian lineage avian H3 viruses. A/canine/Illinois/41915/2015 (H3N2) transmitted by the airborne route at a level of 73–83% in 12 guinea pigs and 1/2 in ferrets. On the other hand, rg-A/canine/Indiana/1117-17-1/2017 (H3N2) did not airborne transmit in ferrets (0/2). Thus, a relatively stable HA protein on its own was insufficient to promote robust airborne transmission of canine H3N2 viruses in ferrets when the viruses had α2,3-receptor specificity.

## 9. H5N1

H5N1 has infected at least 862 humans and caused 455 deaths from 2003 to 2020. Mammalian models of H5N1 pathogenicity have included mice, guinea pigs, ferrets, and non-human primates [188]. In general, the HA proteins of emerging H5N1 isolates have relatively high activation pH values that usually range from pH 5.6 to 6.0 [32,33]. The H5 HA protein has a multi-basic cleavage site that is processed in the secretory pathway by furin-like proteases. Coexpression of the M2 protein (from most any avian or human strain) during trafficking through the mildly acidic trans-Golgi network is needed to prevent premature inactivation of mature HA1/HA2 protein [189].

H5N1 has been passaged in human small airway epithelial (SAE) cells, and HA adaptation mutations have been characterized [190,191]. SAE cell-adapted variants were found to have increased binding to α2,6-linked SA and increases in HA activation pH. Wild-type parental viruses **A/duck/Egypt/D1Br/2007** (H5N1), **A/Shanghai/1/2006** (H5N1; clade 2.3.4), and **A/crow/Kyoto/53/04** (H5N1; clade 2.5) had HA activation pH values of 5.6. The HA1 variants that decreased virion thermostability and increased HA activation pH were the following: pH 5.7 (H1130Y, H130Y/T192I, and H130Y/D158N/T192I); pH 5.8 (A138V/133∆/I155T, S227N, and S227N(I)/133∆/I155T); and pH 5.9 (N186K and N186K/T199I) [190]. An S221P mutation reduced the HA activation pH to 5.3, but the virus containing S221P was outgrown by variants with less stable HA proteins in competition experiments in SAE cells [191]. Thus, growth of H5N1 in SAE cells appears to be promoted by HA destabilization.

A first link between HA stability and IAV pathogenicity was discovered during analyses of 1999 and 2001 avian H5N1 isolates. **A/chicken/Hong Kong/YU562/01 (H5N1)** is highly pathogenic in chickens and has an HA activation pH of 5.7, while **A/goose/Hong Kong/437-10/99 (H5N1)** is only moderately pathogenic and is activated at pH 5.3 [92,192]. X-ray crystal structures show the two HA proteins have similar backbone structures and the stability-altering residues are situated at the HA1-HA1 intermonomer interface in the head (HA1-104 and HA1-115) and at the HA1-HA2 interface between the head and stalk (HA1-216 and HA1-221) [92]. Increases in HA activation pH due to various combinations of these mutations were shown to correlate with increased pathogenicity in chickens [92,192]. For these H5N1 isolates, increases in HA activation pH were shown to increase virulence in chickens.

Amino-acid residues throughout the H5 HA protein, in regions that undergo changes in secondary and tertiary structure upon HA refolding, have been shown to regulate the pH of HA protein conformational changes and activation [88,193]. HA stability mutants of **A/chicken/Vietnam/C58/04 (H5N1)** were studied in cell culture, mallard ducks, and mice (Table 8) [99]. The pH of HA conformational changes and syncytia formation of various mutations were as follows: HA2-N114K (6.4), HA1-Y17H (6.3), WT (5.9), HA1-H18Q (5.6), and HA2-K58I (5.4). All viruses replicated similarly in MDCK cells inoculated at an MOI of 3 PFU/cell. The virus containing HA2-N114K, the least-stable HA protein, was shown to (a) be attenuated in MDCK cells inoculated with 0.01 PFU/cell, (b) be genetically unstable within 5 passages in embryonated chicken eggs, and (c) have low environmental stability at 28 °C. HA1-Y17H was shown to have environmental persistence like WT, while the viruses with stabilizing mutations, HA1-H18Q and HA2-K58I, were longer-lived. Only WT (pH 5.9) and HA1-H18Q (pH 5.6) remained highly pathogenic and transmissible in mallard ducks. Both HA1-Y17H (pH 6.3) and HA2-K58I (pH 5.4) grew to lower levels in the trachea and cloaca, caused no neurological signs or mortality, and did not transmit in mallards. Thus, an optimal HA activation pH in mallards was found to be approximately 5.6–5.9 for these viruses.

The **A/chicken/Vietnam/C58/04 (H5N1)** variants were also studied in mice [195]. The stabilized variant HA2-K58I, which was attenuated in ducks, grew to higher titers in nasal cavities and caused greater weight loss and mortality in DBA/2J mice (Table 8). The destabilizing mutation HA2-Y17H caused attenuation in mice as it had in mallards. This mutation with an activation pH of 6.3 appears to be generally too unstable for in vivo fitness.

The stabilizing HA2-K58I mutation was introduced into **A/Vietnam/1203/04 (H5N1)** HA and rescued in the background of a live-attenuated vaccine candidate that lacks NS1 [196]. HA2-K58I increased vaccine infectivity and immunogenicity in mice. Thus, HA stabilization may enhance live-attenuated influenza vaccines (LAIVs), at least for those containing relatively unstable HA proteins with activation pH values greater than 5.5.

The stabilizing mutation HA2-K58I was also introduced into otherwise-unmodified **A/Vietnam/1203/04 (H5N1)** and infected in ferrets [181]. The stabilizing mutation accelerated virus growth in ferret nasal washes and reduced replication in the lungs. However, a singular HA-stabilizing mutation was unable to promote gain-of-function transmission in ferrets, most likely because this HPAI virus retained a preference to bind α2,3-linked SA receptors. Both HA stabilization and α2,6 receptor-binding in tandem appear necessary for airborne transmissibility in ferrets and human pandemic potential. In summary, H5N1 viruses containing an HA2-K58I mutation that lowered the HA activation pH to 5.4 but retaining avian-preferred α2,3-linked receptor binding were shown to attenuate growth and eliminate transmission in mallards [99] while simultaneously increasing growth in the upper respiratory tracts (URTs) of mice and ferrets [181,195]. Thus, HA stability was shown to play a major role in avian-to-mammalian adaptation of H5N1.

An HA stability mutant of the **A/Vietnam/1194/04 (H5N1)** strain has also been studied in the background of a virus containing the **six internal genes of PR8**, the **NA from A/England/195/2009 (H1N1)**, HA receptor-binding site mutations HA1-Q226L/G228S, and a deleted HA multi-basic cleavage site [180]. In the background of this virus, the parental virus has an HA activation pH of 5.7, while an HA1-H18Q variant is activated at pH 5.2. Compared to an identical virus that lacked the HA1-H18Q mutation, the virus containing the stabilizing HA1-H18Q mutation was shown to enhance growth in the URT and enable contact transmission in one of two co-housed sentinel ferrets. However, despite containing receptor-binding site mutations that increased binding to α2,6-linked SA receptors, the virus with a stabilized HA protein did not airborne transmit in ferrets. This has been speculated to be due, at least in part, to deletion of the HA multi-basic cleavage site [180].

In 2012, two landmark studies were published that showed H5N1 could become airborne transmissible in ferrets with as few as four to six mutations including one that stabilizes the HA protein [51,52]. One study used all eight gene segments from **A/Indonesia/5/2005 (H5N1)** [51], while the other made a 1:7 recombinant with the HA gene from **A/Vietnam/1203/2004 (H5N1**) and the remaining seven genes from **A/California/04/2009 (H1N1)** [52]. The parental viruses in both studies had (a) two receptor-binding site mutations that switched receptor-binding specificity to α2,6-linked SA receptors and (b) the mammalian-preferred PB2-E627K variation. In one study [52], arising first was an HA1-N158D mutation that deleted a glycosylation atop the HA head and enhanced receptor-binding activity. The final mutation needed for gain-of-function airborne transmissibility in ferrets was HA1-T318I in the stalk that decreased the HA activation pH to 5.3 (Table 9). In the other study [51,53], adaptation mutations HA1-T160A (which deleted a glycosylation site in the head) and PB1-H99Y were first detected as minor variants after one passage, while HA1-H110Y (which stabilized head-stalk interactions) was first detected after two passages. HA1-T160A became dominant after three passages and the other two mutations become dominant after seven. Overall, both studies showed that H5N1 required the following properties to become airborne transmissible in ferrets: (a) a mammalian-adapted polymerase, (b) efficient α2,6-linked SA receptor-binding specificity, and (c) an acid-stable HA protein.

The mutations needed for airborne transmission of **A/Indonesia/5/2005 (H5N1)** in ferrets were shown to be attenuating in chickens only when all six were combined [197]. Introduced by itself into the background of the Indo5 virus, the stabilizing HA1-H110Y mutation was only mildly attenuating in chickens. Although, partial reversions to the WT sequence occurred in 1/6 of inoculated and 1/2 contact-transmission chickens, leading the authors to suggest an HA-stabilizing mutation in H5N1 may not be well tolerated in chickens [197].

Differences in HA activation pH have been shown to regulate the cellular tropism of H5N1. Introduction of the HA gene from **A/Hong Kong/483/97 (H5N1)** into a virus containing the other **seven genes from A/California/04/09 (H1N1)** was shown to enable productive replication in RAW264.7 macrophages [165]. Wild-type A/California/04/09 (H1N1) does not replicate in these cells. A subsequent study showed an increase in HA activation pH from 5.4 to 5.9 enables the gain-of-function ability of IAVs to replicate in RAW264.7 macrophages [166].

The HA activation pH values in MDCK cells by A/crow/Kyoto/53/04 (H5N1), A/chicken/Egypt/CL6/07 (H5N1), A/Thailand/Kan353/04 (H5N1), A/Indonesia/5/05 (H5N1), and A/Shanghai/1/06 (H5N1) have been shown to be 5.6–5.7, while those from a panel of avian H2N2, H4N5, H5N3, H5N9, H6N2, H7N7, H8N4 viruses range from pH 5.1–5.5 [198]. The H5N1 viruses with higher HA activation pH values were shown to grow in a larger number of cellular clones of SAE cells transformed with the SV40 large T-antigen. These cells varied in endosomal pH. The avian viruses that had lower HA activation pH values were more restricted in replication. Similar results were obtained in a follow-up study using human tracheal epithelial cell clones (HTEpC-Ts) [199].

Deep sequencing was performed on a panel of **H5N1 viruses isolated from humans** in northern Vietnam from 2004 to 2010 [200]. The most notable changes were in genes that affect receptor binding, polymerase activity, or interferon antagonism. Mutational effects were studied with 7:1 reassortant viruses containing **seven genes from A/California/04/2009 (H1N1)** and the HA gene from human H5N1 isolates. A virus containing the HA gene from **A/Kawasaki/173/2001 (H1N1)** had substantially higher thermostability than a virus containing the HA gene from **A/Vietnam/1203/2004 (H5N1)**. Thermostabilities of minority and majority variants from human H5N1 isolates did not differ. Thus, no HA-stabilizing mutations were identified after H5N1 replication in humans.

## 10. H5Nx

The impact of H5 HA protein stability on antibody recognition and escape has been addressed in several studies. The pH optimum of hemolysis, temperature of HA inactivation, antibody susceptibility, and virulence in mice has been assessed for **H5N1** and **H5N2** HA proteins containing point and combination mutations [201]. **Mouse-adapted A/Mallard/Pennsylvania/10218/84 (H5N2**) was shown to have an HA activation pH of 6.0, and escape mutation HA1-N142K reduced this value to 5.8. Other escape mutations did not affect the pH stability of the HA protein, but several mutations in the HA lateral loop altered thermostability. These studies suggest a disconnect between pH and thermal stability, at least in the context of this H5N2 virus.

A panel of **lentiviral reporter pseudoviruses** containing the HA genes from sixteen H5N1 viruses of different clades/subclades were used to study susceptibility to broadly neutralizing HA stalk-binding antibodies [202]. H5 pseudoviruses causing cell-to-cell fusion at approximately pH 5.3 were less thermostable at 50 °C and more susceptible to broadly neutralizing stalk antibodies FI6, CR6261, and C179 than pseudoviruses with more stable HA proteins with activation pH values of 5.0. Overall, greater HA conformational stability was shown to correlate with reduced binding and neutralization by broadly reactive stalk antibodies in the context of the H5 pseudoviruses.

Highly pathogenic avian **H5N8** emerged in 2014 in migratory birds in North America and reassorted to generate novel **H5N2** viruses [203]. The HA activation pH and virus inactivation pH values of four H5N8 and seven H5N2 isolates have been measured to range from 5.9 to 6.0 [204]. The viruses were also found to bind preferentially to α2,3-linked SA receptors. The viruses grew only to low levels in the nasal cavities of inoculated ferrets and did not transmit to contact ferrets. Thus, the North American H5Nx viruses appeared poorly adapted for infection in ferrets and, presumably, humans.

Adaptation of **A/duck/Liaoning/LN/2011(H5N5)** to mice resulted in virulence mutations PB2-E627K and HA2-F110L, which decreased MLD_50_ values in mice 1000-fold and 3.16-fold, respectively [205]. Based on the position of HA2-F110L in the hinge region of the central triple-stranded core of the HA protein, the mutation has been speculated to alter HA stability. However, follow-up studies are needed to confirm this hypothesis and to determine an optimal HA activation pH for H5N5 virulence in mice.

**A/Guangzhou/39715/2014 (H5N6)** contains the mutation PB2-E627K, has enhanced polymerase activity in mammalian cells, and causes greater URT growth and more severe pneumonia in ferrets than previously investigated clade 2.3.4.4 H5 viruses [206]. A/Guangzhou/39715/2014 (H5N6) was shown to bind preferentially to avian-preferred α2,3-linked receptors and to induce syncytia at a relatively high value of pH 5.6. However, this virus does not airborne transmit between ferrets. Thus, this human clade 2.3.4.4 H5N6 virus does not have known mammalian adaptation markers that are thought to be needed for human pandemic potential.

## 11. H7Nx

The virus inactivation pH values of highly pathogenic fowl plague viruses (FPVs) **A/FPV/Rostock/34 (H7N1)** and **A/FPV/Dutch/27 (H7N7)** have been measured to be 5.8 and 5.6, respectively [34]. Those of **five other LPAI H7 viruses** range from 5.6 to 6.0. When expressed alone in CV-1 cells, **A/FPV/Rostock/34 (H7N1)** has been shown to be protected from inactivation in the mildly acidic secretory pathway by coexpression of the M2 protein and to be protected from inactivation in the presence of ammonium chloride or high concentrations of amantadine, which raise intracellular pH [207].

The highly pathogenic virus **A/ostrich/Italy/2332/2000 (H7N1)** gained airborne transmissibility in ferrets after 10 serial passages [208]. Mutations conferring airborne transmissibility in ferrets were identified as PB2-T81I, NP-V284M, M1-R95K, M1-Q211K, and HA1-K/R304R. HA1 residue 304 is situated near the top of the stalk, where it interacts with the HA2 B-loop, and is distal to the receptor-binding site. Based on the HA protein structure, the mutation at HA1-304 has been speculated to alter HA stability. Follow-up experiments were curtailed due to regulatory policies but would be needed to confirm whether HA stabilization enhanced the airborne transmissibility of H7N1 in ferrets like what has been observed for ferret adapted H5N1 viruses.

LPAI H7N2 caused an outbreak in a cat shelter in New York City in December 2016 [209]. **A/New York/108/2016 (H7N2)**, a virus transmitted to a human, was found to cause mild, transient illness in ferrets but did not transmit by the airborne route [210]. A/New York/108/2016 (H7N2) was shown to induce syncytia formation at pH 5.4, a lower value than other H7 viruses including **A/New York/107/2003 (H7N2)** (5.8), **A/Turkey/Virginia/4529/2002 (H7N2)** (5.9) and **Anhui/1/2003 (H7N9)** (5.8). A/New York/108/2016 (H7N2) has a deleted 220 loop (which contains HA1 residues 226 and 228) and binds to both α2,3- and α2,6-linked SA receptors. Overall, these H7N2 viruses do not appear to be fully adapted to ferrets and, in their present form, are thought to pose a limited risk for human pandemic potential.

## 12. H7N9

H7N9 is first known to infect humans in China in February 2013 [211]. By March 2021, a total of 1,568 human infections with H7N9 have been reported to the WHO. Five major waves of human infections of H7N9 occurred between 2013 and 2017, and the successful application of a bivalent H5/H7 vaccine prevented subsequent chicken infections, and as a result, nearly eliminated human infections [212].

Several risk assessment studies were done on H7N9. Multiple studies showed first-wave virus **A/Anhui/1/2013 (H7N9)** has only limited airborne transmission in ferrets and guinea pigs [194,213,214,215]. In one study, A/Anhui/1/2013 was shown to induce syncytia at pH 5.6 [214]. Adaptation to ferrets yielded a double mutant HA1-N123D/N149D, which enhanced α2,6-linked SA receptor binding but destabilized the HA protein by lowering the activation pH to 5.8 and reducing thermostability. Overall, these mutations were not considered to increase the risk of the virus for airborne transmissibility. In a follow-up study, stabilizing HA mutations that might promote adaptation of H7N9 viruses to mammals were investigated [216]. HA1-N104K, which is in the 110 helix at the HA1-HA2 interface, was found to decrease the HA activation pH to 5.2, and HA2-K58I, located in HA2 helix A that packs into the HA2 coiled-coil core, decreased the HA activation pH to 5.0. These two mutations were also shown to increase the thermostability of A/Anhui/1/2013.

In another study, **A/Shanghai/1/2013** had an HA activation pH of 5.6, while **A/Anhui/1/2013** and **A/Shanghai/2/2013** had HA activation pH values of 5.8 [194]. Infection of A/Anhui/1/2013 in chickens resulted in many HA sequence variants while infection in ferrets produced no major variations in the HA gene, showing adaptation of H7N9 to ferrets may have a narrow genetic bottleneck.

Transfected **A/Anhui/1/2013** HA gene has been shown to induce syncytia formation and cell–cell fusion by luciferase reporter assay at pH 5.8 [213]. It has also been shown to be susceptible to proteolysis at a pH between 6.03 and 6.59 [217].

After first-wave H7N9 viruses were shown to have relatively unstable HA proteins and were poorly airborne-transmissible in ferrets and guinea pigs, second- and third-wave H7N9 viruses were characterized [218]. In hemolysis assays, **A/Anhui/1/2013 and other first- and second-wave isolates** had HA activation pH values of pH 5.8. Third-wave viruses **A/British Columbia/1/2015** and **A/Hong Kong/56/2015** had marginally stabilized HA proteins that induce hemolysis at 5.6. When infected in ferrets, third-wave H7N9 viruses transmitted efficiently to contact ferrets but had limited transmissibility by the airborne route.

Fifth-wave viruses characterized include three LPAI viruses (**A/Hong Kong/4553/2016, A/Hong Kong/125/2017**, and **A/Hong Kong/61/2016**) and two HPAI viruses (**A/Guangdong/17SF003/2016** and **A/Taiwan/1/2017**) [219]. In syncytia assays, A/Hong Kong/61/2016 had an activation pH 5.8, and the other two LPAI viruses had HA activation pH values of 5.7. The two HPAI fifth-wave viruses studied had stabilized HA proteins that have HA activation pH values of 5.4. Such an enhanced stability has been associated with adaptation to ferrets and humans. Overall, the fifth-wave H7N9 viruses caused enhanced disease in mice and ferrets but had a limited capability for airborne transmission in ferrets.

**A/Anhui/1/2013 (H7N9)** has been serially passaged in the presence of ferret antiserum, resulting in escape mutants HA1-A125T, HA1-A151T, and HA1-L217Q [220]. These residues are in and around the receptor-binding pocket. The HA1-L217Q mutation was shown to decrease the pH of syncytia formation by 0.1 pH units and increase the inactivation temperature from 50 to 56.1 °C. Overall, the escape mutants (a) enhanced growth in cell culture and embryonated chicken eggs, (b) increased acid and thermal stabilities by a small extent, and (c) reduced binding to α2,6-linked SA receptors while maintaining binding for avian-like α2,3-linked SA receptors. Thus, the major effects of the escape variants were substantial changes in receptor binding, not HA stability.

## 13. H9N2

An avian H9N2 virus, A/turkey/Wisconsin/1966 (H9N2), was first isolated during an outbreak in February, 1966, in northern Wisconsin [221]. H9N2 viruses continue to circulate in North America in turkeys and wild birds at a low frequency [222,223]. Eurasian H9N2 viruses of G1 (A/quail/Hong Kong/G1/1997) and Y280/G9 (A/chicken/Beijing/1/1994) lineages have become endemic in Asia, the Middle East, and Northern Africa [224,225,226]. Human cases of H9N2 have been reported since 1999 [227], prompting a number of risk assessment studies on H9N2 viruses [228,229].

Three large-scale risk assessment studies have been performed that have characterized the HA activation pH values of H9N2 viruses from both G1 and Y280 lineages. In one study, a diverse set of eleven **H9N2 viruses isolated during** the period **1994–2009 from a duck, a guinea fowl, chickens, swine, humans, and the environment** were assessed for pandemic risk, and none airborne transmitted in ferrets [230]. The HA activation pH values of these viruses ranged from pH 5.3 to 5.7 except for the outlier **A/guinea fowl/Hong Kong/NT101/2003** (activation pH 4.8) [33]. In a second study, **17 H9N2 viruses isolated from quails, chickens, passerine, turkeys, and humans in** the period **1966–2011** were also shown to have HA activation pH values that ranged from pH 5.4 to 5.8 [231]. In a third study, HA activation pH values ranged from pH 5.3 to 5.8 for **5 human and 4 chicken H9N2 isolates** [232]. In this third study, the airborne transmissibilities of three isolates were investigated. **A/Hong Kong/308/2014** (B Y280 lineage) with an activation pH of 5.4–5.5 was moderately transmissible, while **A/Hong Kong/1073/1999** (G1 lineage) with a destabilized HA protein (activation pH 5.8) was not airborne transmissible. Overall, **A/Anhui-Lujiang/39/2018** (B Y280 lineage), which has an HA activation pH of 5.4, had greater replication and airborne transmissibility in ferrets than the other isolates. This recent study highlights the ongoing threat of H9N2 viruses to gain human pandemic potential, especially viruses such as A/Anhui-Lujiang/39/2018.

**A/Chicken/Shandong/Li-2/2010 (H9N2)** was passaged 15 times in guinea pigs [233]. This H9N2 virus gained airborne transmissibility in guinea pigs by acquiring mutations HA1-Q227P, HA2-D46E, and NP-E434K. The HA1-Q227P and HA2-D46E substitutions were shown to enhance binding to both avian- and human-type receptors, while HA2-D46E increased virus thermostability at 50–58 °C. The NP-E434K mutation was shown to enhance viral RNA polymerase activity in vitro. HA activation pH values for the parental and adapted viruses were not described; however, enhanced HA thermostability was associated with transmissibility in guinea pigs.

**A/chicken/Shanghai/7/2001 (SH7)** was found to be airborne transmissible in chickens, whereas the closely related strain **A/chicken/Shanghai/14/2001 (SH14)** was not [234]. Using a panel of reassortant viruses, residues HA-K363 and PA-L672 were found to enable the airborne transmissibility of SH7 among chickens. In particular, the SH7 HA-K363 variation was shown to increase resistance of H9N2 virions to acid inactivation. The virus inactivation pH of values of the airborne-transmissible SH7 and non-transmissible SH14 viruses were shown to be 5.0 and 5.9, respectively. Thus, the more transmissible H9N2 virus in chickens was more acid stable.

**A mouse-adapted variant of A/swine/Hong Kong/9/98 (H9N2)** was serially passaged in the presence of a panel of H9 mAbs to generate escape variants [235]. The parental virus had a temperature of inactivation of 57.5 °C, while individual escape mutants containing HA1 mutations S133N, T189A, N198D, and L226Q had inactivation temperatures raised to 59.3 °C. While this difference is small, it was calculated to be statistically significant. Values for the pH of hemolysis of the parental and escape variants were approximately 5.8. Overall, selection of H9N2 escape variants resulted in less substantial effects on HA stability than an analogous study previously performed on an H7N9 virus [201,235], and these small changes in thermostability did not substantially alter pH stability.

## 14. H10N7

A 2014 outbreak of H10N7 influenza viruses caused high mortality in seals in northwestern Europe [236]. In one study [237], the HA sequences from 26 H10N7 seal viruses isolated in 2014 and 2015 were found to differ from H10Nx avian viruses isolated in the period 1949–2015 by nine amino-acid variations in HA1: E91K, S122N, T171A, Q210K, N212S, Q226L, N242K, T244I, and M327V. To understand the importance of these variations, a panel of recombinant viruses were generated and characterized including **rg-A/turkey/England/384/79 (H10N4)**, a 7:1 virus containing seven genes from H10N4 and the HA from **A/seal/Germany/AR2351/1/14 (H10N7)**, and eight H10N4 viruses containing all the aforementioned HA variations except T171A. The H10N4 HA protein bound preferentially to α2,3-linked SA receptors, the H10N7 HA protein bound both α2,3- and α2,6-linked SA receptors, and the Q226L mutation enhanced binding to α2,6-linked SA receptors and abrogated binding to α2,3-linked SA receptors. In the background of H10N4, the Q210K, N212S, and N242K mutations were shown to increase virus thermostability at 56 °C compared to wild type. Overall, several of the HA traits that have arisen in seals are those that have been shown to enhance adaptation to other mammals such as α2–6 receptor-binding specificity and HA stability.

A second study comprehensively dissected the molecular traits associated with the airborne transmissibility of H10N7 viruses in ferrets [238]. Avian strain **A/mallard/NL/1/2014 (H10N7)** airborne transmitted in 1/8 ferrets, the early seal isolate **A/harbor seal/Germany/1/14 (H10N7)** transmitted in 1/4 ferrets, and the late seal isolate **A/harbor seal/Netherlands/PV14-221_TS/2015** transmitted in 6/8 ferrets. Thus, the Netherlands (NL) was highly transmissible, and this virus was shown to preferentially bind α2,6-linked SA receptors on long extended branches. The two less transmissible isolates preferentially bound to α2,3-linked SA. Using syncytia assays, the authors showed that the more transmissible NL virus was activated for membrane fusion at pH 5.2, a relatively stable value, while the less transmissible mallard virus was activated at pH 5.5. Twenty recombinant HA mutants were generated in the background of the mallard virus and five in the background of the NL seal virus. Introducing either the HA1-T244I and HA2-E74D variations into the mallard virus were found to stabilize its HA protein by 0.2 units, while the opposite mutations in the background of the HA from the NL virus (HA1-I244T or HA2-D74E) were destabilizing by 0.2 pH units. Thus, these two HA protein substitutions have been shown to increase the stability of the seal H10 virus compared to the avian virus such that the HA activation pH of the more airborne-transmissible NL H10N7 virus has the relatively stable value of 5.2. Overall, the HA protein of the seal NL virus was shown to bind preferentially to α2,6-linked SA and to be highly stabilized, two markers shown to be necessary for human adaptation.

## Figures and Tables

**Table 1:** Phenotypes of seasonal and pandemic H1N1 variants in vitro.

Strain	Activation pH	Phenotype	Reference
A/Hamburg/04/2009 HA2-I91L	5.9	↑ growth A549	[145]
A/Hamburg/04/2009 WT	5.7	--	
(A/Ann Arbor/6/60 *ca*) A/California/7/2009 HA WT	5.5	--	[146]
(A/Ann Arbor/6/60 *ca*) A/California/7/2009 HA2-E47K	5.3	↓ growth Vero, = growth MDCK	
(A/Ann Arbor/6/60 *ca*) A/Brisbane/10/2010 HA2-K47E	5.5	↑ growth Vero, = growth MDCK	[146]
(A/Ann Arbor/6/60 *ca*) A/Brisbane/10/2010 HA WT	5.3	--	
A/Puerto Rico/8/34 HA2-N117D	5.4	↑ growth Vero, = growth MDCK	[143]
A/Puerto Rico/8/34 WT	5.2	--	

**Table 2:** Phenotypes of seasonal and pandemic H1N1 variants in mice.

Strain	Activation pH	Phenotype	Reference
A/Tennessee/1-560/2009 HA1-Y17H	6.0	↓↓ virulence in mice	[152,154]
A/Tennessee/1-560/2009 WT	5.5	--	
A/Tennessee/1-560/2009 HA2-R106K	5.3	↓ virulence in mice	
A/England/195/2009 HA1-Y17H	5.9	↓↓ virulence in mice	[153]
A/England/195/2009 HA1-A19T	5.8	↓ virulence in mice	
A/England/195/2009 WT	5.5	--	
A/England/195/2009 HA1-E31K	5.3	↓ virulence in mice	
A/FM/1/47	5.7	--	[147]
A/FM/1/47 HA2-W47G	5.5	↑ virulence in mice	
A/Puerto Rico/8/34 HA1-P78L, HA2-H25Q	5.5	↑ virulence in mice	[144]
A/Puerto Rico/8/34	5.3	--	
A/WSN/33 (H1N1) WT	5.4	--	[148]
A/WSN/33 (H1N1) HA2-T64H, HA2-V66H	5.2	↓ virulence in mice	

**Table 3:** Phenotypes of A/Tennessee/1-560/2009 (H1N1) and A/England/195/2009 (H1N1) HA stability variants in vitro.

Strain	Activation pH	Low-MOI Phenotype	High-MOI Phenotype	Reference
TN09 HA1-Y17H	6.0	↓ growth mNEC, mTEC	↑ growth MDCK, A549, RAW 264.7	[152,154]
TN09 WT	5.5		--	
ENG09 HA1-Y17H	5.9	↓↓ growth HAE	↑ growth MDCK, A549	[153]
ENG09 HA1-A19T	5.8	↓ growth HAE	↑ growth MDCK, A549	
ENG09 WT	5.5	--	--	
ENG09 HA1-E31K	5.3	= growth HAE	= growth MDCK, A549	

**Table 4:** A/Tennessee/1-560/2009 (H1N1) HA stability variants in ferrets [152].

Virus	Activation pH	Nasal Growth in Inoculated	Airborne Transmission	Variant Transmission	Transmitted Variant
TN09 HA1-Y17H	6.0	↓ growth; delayed	0/4	1/4	HA1-H17Y/HA2-R106K (pH 5.3)
TN09 WT	5.5	--	4/4	--	--

**Table 5:** A/England/195/2009 (H1N1) HA stability variants in ferrets [169].

Virus	Activation pH	Nasal Growth in Inoculated Ferrets	Delay in Peak Shedding	Air-Emitted Virus Plaques	Variants in Donor Ferrets and in Emitted Virus Recovered
ENG09 HA1-Y17H	5.9	Similar A.U.C.	1 day	23	HA1-H17Y, HA2-V55I, HA2-E47K, HA1-V29I
ENG09 HA1-E31K	5.3	Similar A.U.C.	--	184	--

**Table 6:** A/Tennessee/1-560/2009 (H1N1) HA stability variants in swine and co-housed ferrets [173].

Virus	Activation pH	Growth in Inoculated Swine	Contact Transmission to Swine	Airborne Transmission to Ferrets	Variants Transmitted to Ferrets	Transmitted Variants
TN09 HA1-Y17H	6.0	↓ growth; delayed	3/3; delayed	0/3	3/3	HA1-H17Y, HA2-V55I, HA2-R106K
TN09 WT	5.5	--	3/3	3/3	--	--
TN09 HA2-R106K	5.3	WT-like	3/3	3/3	--	--

**Table 7:** A/swine/Illinois/2A-1213-G15/2013 (G15) HA stability variants in ferrets [176].

Virus	Activation pH	% of Inoculum	% 3d Post infection	Airborne Transmitted
A/sw/IL/2A-1213-G15/2013 HA1-N210	5.8	~85%	<10%	0/3
A/sw/IL/2A-1213-G15/2013 HA1-S210	5.5	~15%	>90%	3/3

**Table 8:** A/chicken/Vietnam/C58/04 (H5N1) HA stability variants in mallards and mice [99,194].

Virus	Activation pH	Environmental Persistence, 28 °C	Growth, Morbidity, and Mortality in Mallards	Transmission in Mallards	Growth, Morbidity, and Mortality in Mice
CH58 HA1-Y17H	6.3	61 d	↓	↓	↓
CH58 WT	5.9	62 d	--	--	--
CH58 HA1-H18Q	5.6	77 d	WT-like	WT-like	WT-like
CH58 HA2-K58I	5.4	79 d	↓	↓	↑

**Table 9:** Phenotypes of A/California/04/2009 (H1N1) viruses bearing A/Vietnam/1203/04 (H5) HA1 variants in ferrets [52].

Virus	Activation pH	Receptor-Binding Specificity	Contact Transmission in Ferrets	Airborne Transmission in Ferrets	Transmitted Variants
WT	5.6	α2,3	0/3	0/3	--
N224K/Q226L	5.8	α2,6	0/3	0/3	--
N158D/N224K/Q226L	5.8	α2,6	5/6	0/6	2/6 (T318I)
N158D/N224K/Q226L/T318I	5.4	α2,6	5/6	4/6	--
